# From Secondary Hyperparathyroidism to Neurologic Crisis: A Tale of Brown Tumor and Spinal Cord Compression

**DOI:** 10.1210/jcemcr/luaf022

**Published:** 2025-03-20

**Authors:** Aryn Kormanis, Matthew Anderson, Miya McKnight, Hima Darapu

**Affiliations:** Department of Endocrinology, Atrium Health Wake Forest Baptist Medical Center, Winston-Salem, NC 27157, USA; Department of Endocrinology, Atrium Health Wake Forest Baptist Medical Center, Winston-Salem, NC 27157, USA; Department of Endocrinology, Atrium Health Wake Forest Baptist Medical Center, Winston-Salem, NC 27157, USA; Department of Endocrinology, Atrium Health Wake Forest Baptist Medical Center, Winston-Salem, NC 27157, USA

**Keywords:** brown tumor, secondary hyperparathyroidism, ESRD, thoracic spine, parathyroidectomy, cord compression

## Abstract

A 26-year-old male with medical history of polycystic kidney disease, IgA nephropathy, end-stage renal disease, and brown tumor (requiring prior surgical intervention on brown tumor) was hospitalized because of right lower extremity weakness. Examination revealed right hip flexor weakness and right foot clonus. Laboratory results showed serum calcium of 10.4 mg/dL (2.59 mmol/L) (reference range: 8.5-10.5 mg/dL; 2.12-2.62 mmol/L). Magnetic resonance imaging displayed a 2.5 × 3.7 cm soft tissue mass with circumferential encroachment of the epidural space leading to severe spinal canal stenosis of T4-T5 at the site of prior brown tumor resection. The patient underwent subtotal parathyroidectomy with left cervical thymectomy, leading to an intraoperative PTH drop from >3500 pg/mL (>371 pmol/L) (reference range: 12-88 pg/mL; 1.27-9.33 pmol/L) to 247 pg/mL (26.182 pmol/L). In the context of treating vertebral brown tumors, the consensus suggests that parathyroidectomy plays a crucial role in achieving both clinical and radiographical amelioration of the tumor. However, in cases like ours where paresis is present, surgical decompression of the tumor becomes imperative. The development of brown tumors is an uncommon and severe, long-term complication for hyperparathyroidism. The location of a brown tumor in the thoracic spine causing symptomatic cord compression is rare.

## Introduction

Secondary hyperparathyroidism (SHPT) is a common complication of end-stage renal disease (ESRD). Here we present a rare and severe consequence of SHPT—a brown tumor leading to spinal cord compression, in a young adult with ESRD. Our case is among the very few reports in the literature emphasizing the rarity and complexity of a recurrent brown tumor causing spinal cord compression, and it underscores the critical role of timely intervention in preventing permanent neurologic defects.

## Case Presentation

A 26-year-old male with a medical history of polycystic kidney disease and IgA nephropathy complicated by ESRD (diagnosed in 2016 and started on hemodialysis), hypertension, anemia of chronic disease, calciphylaxis, SHPT, and brown tumors (with prior surgical intervention) was admitted for acute lower extremity paralysis in the setting of SHPT after presenting to the emergency department.

In 2022, 6 years following diagnosis of ESRD, he developed SHPT with the onset of brown tumors. At that time, he underwent T5 laminectomy with tumor debulking and T4-T6 fusion due to lower extremity weakness as sequelae of the brown tumors. During this presentation, he had 3/5 motor strength in the left lower extremity and 0/5 strength in the right lower extremity. Imaging with computed tomography showed numerous lucent osseous lesions in the thoracic spine, the largest of which was 3.8 cm at T5 with substantial epidural extension, resulting in severe spinal canal narrowing and cord compression prompting laminectomy and vertebral fusion. On laboratory evaluation, he had an intact PTH (iPTH) level that exceeded 3500 pg/mL (>371 pmol/L) (normal reference range: 12-88 pg/mL; 1.27-9.33 pmol/L), vitamin D (OH) was low at ng/mL (37.44 nmol/L) (normal reference range: 30-100 ng/mL; 74.88-249.6 nmol/L), vitamin D 1,25(OH)_2_ was also low at 5.7 pg/mL (14.23 pmol/L) (normal reference range: 24.8-81.5 pg/mL; 61.9-203.42 pmol/L), phosphorus was elevated at 6.7 mg/dL (2.16 mmol/L) (normal reference range 2.5-5 mg/dL; 0.807-1.615 mmol/L), and a corrected calcium level was 7.8 mg/dL (1.95 mmol/L) (normal reference range: 8.5-10.5 mg/dL; 2.12-2.62 mmol/L) (Table 1). Though amenable to vertebral laminectomy with debulking and vertebral fusion, he declined parathyroidectomy and was instead treated medically with calcitriol 0.25 mcg daily, ergocalciferol 50 000 units weekly, and sevelamer 3200 mg 3 times daily with meals. No postoperative advanced imaging was obtained before discharge and so further radiologic imaging of canal stenosis was not available at time of subsequent hospitalization; however, he was noted to have improved strength at discharge with ability to raise both lower extremities against gravity. He was lost to follow up at the time of discharge; additionally, he had no further laboratory work available for review on readmission.

**Table 1.** Laboratory markers at time of initial diagnosis of brown tumor, during subsequent hospital admission before parathyroidectomy, and postparathyroidectomy

Laboratory test	Normal range	At initial diagnosis (4 months before current admission)	Current admission (before parathyroidectomy)	Current admission (postparathyroidectomy)	Follow-up
Serum calcium	8.5-10.5 mg/dL; 2.1-2.6 mmol/L	7.8 mg/dL; 2.0 mmol/L	10.0 mg/dL; 2.5 mmol/L	7.7 mg/dL; 1.9 mmol/L	7.9 mg/dL; 2.0 mmol/L
Phosphorus	2.5-5.0 mg/dL; 0.8-1.6 mmol/L	6.7 mg/dL; 2.2 mmol/L	5.0 mg/dL; 1.6 mmol/L	2.9 mg/dL; 0.9 mmol/L	NA
Intact PTH	12-88 pg/mL; 1.3-9.3 pmol/L	>3500.0 pg/mL; >9.3 pmol/L	>3500.0 pg/mL; >9.3 pmol/L	NA	443.0 pg/mL; 47.0 pmol/L
Vitamin D 25(OH)	30-100 ng/mL; 74.9-249.6 nmol/L	15.0 ng/mL; 37.4 nmol/L	19.0 ng/mL; 47.2 nmol/L	NA	35.0 ng/mL; 87.4 nmol/L
Vitamin D 1,25(OH)_2_	24.8-81.5 pg/mL; 61.9-203.4 pmol/L	5.7 pg/mL; 14.2 pmol/L	NA	NA	NA
Corrected calcium	8.5-10.5 mg/dL; 2.1-2.6 mmol/L	NA	10.4 mg/dL; 2.6 mmol/L	NA	7.3 mg/dL; 1.8 mmol/L
Alkaline phosphatase	34-104 U/L; 0.6-1.7 µkat/L	NA	NA	NA	NA

Abbreviation: NA, not available.

On current presentation, he returned with worsening right lower extremity weakness and new right hip flexor weakness with magnetic resonance imaging (MRI) showing tumor recurrence at T5.

## Diagnostic Assessment

**Figure 1.**
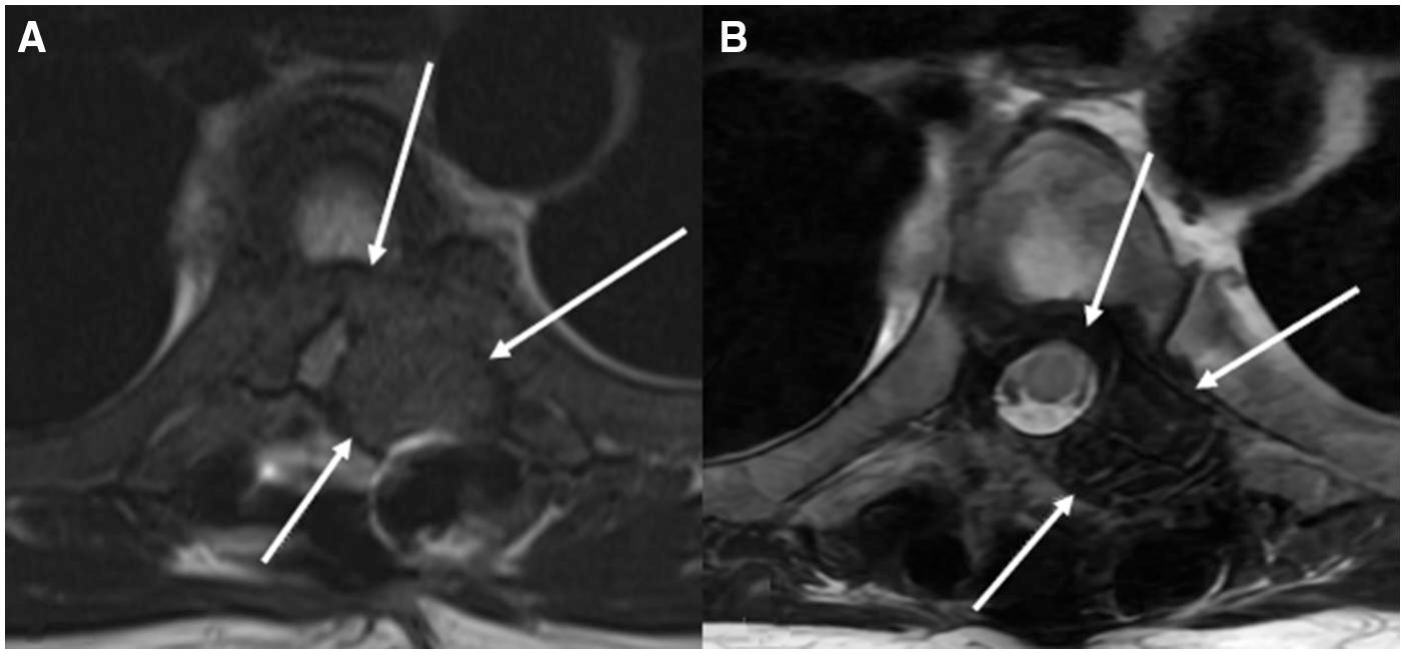
Magnetic resonance imaging (MRI) of the thoracic spine demonstrating prior T4-T6 posterior fusion with T5 laminectomy and T5 tumor debulking with interval worsening of 2.5 × 3.7 cm soft tissue neoplasm resulting in severe canal stenosis and cord compression/edema with near-complete collapse of the T5 vertebral body (A). MRI of the thoracic spine obtained 7 months after parathyroidectomy, demonstrating significant decrease in the size of the soft tissue tumor at T5 without signs of spinal canal stenosis (B).

During this presentation, examination revealed new right 4/5 hip flexor weakness and right foot 3-beat clonus. Laboratory results showed serum calcium of 10.4 mg/dL (2.59 mmol/L), phosphorus of 5.8 mg/dL (1.87 mmol/L), and vitamin D 25(OH) of 19 ng/mL (47.24 nmol/L) (Table 1). He reported medication nonadherence to the previously recommended calcitriol, ergocalciferol, and sevelamer. MRI displayed a 2.5 × 3.7 cm soft tissue mass with circumferential encroachment of the epidural space leading to severe spinal canal stenosis of T4-T5 (Fig. 1A), worse than in previous imaging. Orthopedics recommended supportive care with cervical thoracic orthotics brace based on neurologic examination.

## Treatment

Due to the patient's neurologic deficits with progression of brown tumor growth, subtotal parathyroidectomy was suggested at this juncture and further spinal surgical intervention was deferred. The patient underwent subtotal parathyroidectomy with left cervical thymectomy.

## Outcome and Follow-up

During surgical intervention, the patient experienced an intraoperative PTH level drop from >3500 pg/mL (>371 pmol/L) to 332.8 pg/mL (35.28 pmol/L). Postoperatively, he developed hungry bone syndrome with a postoperative ionized calcium drop as low as 0.95 mmol/L (0.95 mmol/L) (normal reference range: 1.15-1.33 mmol/L; 1.15-1.33 mmol/L). He had a phosphorus level of 3.4 mg/dL (1.1 mmol/L) and alkaline phosphatase of 697 U/L (11.62 µkat/L) (normal refence range: 34-104 U/L; 0.57-1.73 µkat/L) (Table 2). Given electrolyte derangements, he needed ongoing calcitriol and calcium carbonate supplementation. On outpatient orthopedic follow-up, the patient was noted to have improved right hip flexor muscle strength and improvement in the size of the T5 mass on repeat MRI 7 months after parathyroidectomy (Fig. 1B).

**Table 2.** Intraoperative and postoperative laboratory markers during and following parathyroidectomy

Laboratory test	Normal range	Intraoperative (parathyroidectomy)	Postoperative day 1	Postoperative day 2	Postoperative day 3
Serum calcium	8.5-10.5 mg/dL; 2.1-2.6 mmol/L	NA	7.2 mg/dL; 1.8 mmol/L	6.5 mg/dL; 1.6 mmol/L	7.2 mg/dL; 1.8 mmol/L
Phosphorus	2.5-5.0 mg/dL; 0.8-1.6 mmol/L	NA	3.3 mg/dL; 1.1 mmol/L	3.4 mg/dL; 1.1 mmol/L	3.7 mg/dL; 1.2 mmol/L
Intact PTH	12-88 pg/mL; 1.4-9.3 pmol/L	332.8 pg/mL; 35.3 pmol/L	NA	NA	NA
Corrected calcium	8.5-10.5 mg/dL; 2.1-2.6 mmol/L	NA	7.4 mg/dL; 1.9 mmol/L	6.7 mg/dL; 1.7 mmol/L	7.6 mg/dL; 1.9 mmol/L
Alkaline phosphatase	34-104 U/L; 0.6-1.7 µkat/L	NA	666.0 U/L; 11.1 µkat/L	697.0 U/L; 11.6 µkat/L	690 U/L; 11.5 µkat/L

Abbreviation: NA, not available.

## Discussion

SHPT is a complex consequence of chronic kidney disease, characterized by disrupted calcium and phosphorus homeostasis and elevated iPTH levels. In chronic kidney disease, impaired renal function leads to hyperphosphatemia, low serum calcium, and reduced activation to 1,25(OH)_2_ vitamin D. These factors together contribute to the overstimulation of iPTH secretion, resulting in secondary hyperparathyroidism. Prolonged hyperparathyroidism can lead to down regulation of vitamin D and calcium receptors, posing challenges for effective medical management of this condition [1].

In this case, the patient's SHPT was initially managed with calcitriol and phosphate binders; however, poor adherence to treatment and loss of follow-up led to inadequate SHPT control.

Persistently elevated PTH levels (>3500 pg/mL, > 371 pmol/L) caused ongoing bone resorption, contributing to the formation and recurrence of brown tumors. The overproduction of PTH increases receptor activator of nuclear factor kappa-B (RANK) ligand activity, stimulating osteoclasts and leading to bone resorption, cortical bone destruction, and the formation of fibrous cysts. This process also produces osteoclast-like giant cells and fibrous tissue that replace bone marrow, resulting in brown tumors [2].

The failure to suppress PTH and correct the calcium-phosphorus imbalance maintained high bone turnover, allowing the brown tumor to grow and compress the spinal cord. This case highlights the critical role of effective SHPT management, through either sustained medical therapy or timely parathyroidectomy in preventing such severe complications.

The overproduction of PTH leads to increased RANK ligand activity via osteoblasts allowing for increased attachment to RANK on osteoclasts. Subsequently, there is bone resorption, cortical bone destruction, and fibrous cyst formation. This may also lead to the production of osteoclast-like giant cells and fibrous tissue replacing the bone marrow, which is very proliferative, and otherwise known as a brown tumor [3, 4]. On pathological evaluation, the brown tumor contains giant bone cells, spindle stromal cells, and hemosiderin granules, which give the tumor its characteristic brown appearance [5].

As of 2021, the prevalence of brown tumors in SHPT was documented to be 13% [6]. Notably, as of 2013 there were 30 documented vertebral brown tumors leading to neurologic compromise. Only 16 were associated with SHPT [7]. These prevalence rates illustrate not only the seriousness, but also the rarity of vertebral brown tumors as a complication. This case emphasizes the potential severity of untreated or refractory SHPT in ESRD patients. Of the 16 documented neurologically compromising vertebral brown tumors associated with SHPT, 10 were located within the thoracic spine. Eight of the 10 thoracic brown tumors underwent resection of the tumor and only 2 had documented resolution of symptoms, though multiple had some level of improvement, and the outcomes of others were not reported [7].

We identified a relevant case report of thoracic spine brown tumor in a patient in whom surgical intervention was initially deferred as she opted for medical management for 4 years until onset of neurologic symptoms. Of note, she was on peritoneal dialysis rather than hemodialysis, though any relevant role related to such is unclear. Despite surgical intervention by neurosurgery, at the time of the published case report, her extremity motor function had not fully recovered [8].

These outcomes highlight the uniqueness of our case: not only did our patient encounter a recurrent vertebral tumor after initial debulking, but also experienced subsequent paresis resulting from cord compression, from which he was able to undergo successful rehabilitation and regain complete motor strength after neurosurgical intervention.

In the context of treating vertebral brown tumors, the consensus suggests that parathyroidectomy plays a crucial role in achieving both clinical and radiographical amelioration of the tumor [9]. However, in cases like ours where paresis is present, surgical decompression of the tumor becomes imperative. Bisphosphonate therapy can also be added for additional bone protection by reducing osteoclast activity.

After parathyroidectomy, our patient developed hungry bone syndrome, a known complication in dialysis patients, with an occurrence rate of approximately 25% [10]. This condition is characterized by rapid shifts of calcium and phosphorus from the bloodstream into the bones, necessitating close postoperative monitoring and aggressive supplementation of calcium and vitamin D [11]. Our case again underscores the importance of vigilant monitoring for electrolyte imbalances and timely intervention to manage this common complication.

## Learning Points

- The development of brown tumors is a severe and rare long-term complication for hyperparathyroidism.
- The location of a brown tumor in the thoracic spine causing symptomatic cord compression is even more rare in our review.
- Interdisciplinary management of hyperparathyroidism including endocrinology, nephrology, orthopedic, and endocrine surgery is of the utmost importance for timely surgical intervention to prevent permanent neurologic deficits and, ultimately, effective management of complex cases like this one.

## Data Availability

Data sharing is not applicable to this article as no datasets were generated or analyzed during the current study.

## Abbreviations

ESRD: end-stage renal disease
iPTH: intact PTH
MRI: magnetic resonance imaging
RANK: receptor activator of nuclear factor kappa-B
SHPT: secondary hyperparathyroidism
